# Smartweed

**Published:** 1878-09

**Authors:** 


### Smartweed.

The smartweed, "Polygonum Punctatum," P. hydropiperoide, is a common weed, well known as a domestic remedy; but its true place in therapeutics is but little known or valued. In disentery, watery, and mucous diarrhœa, few agents have proven of more value. The dry plant yields about eighteen per cent. of tannin. What its other proximal principles are I do not know. I prepare a saturated tincture. As an anti-pyretic in typhomalarial and our bilious remittent fevers, it has few equals. With it the temperature may, in a few hours, be brought down from 102°, or even 104°, to 98°, and at the same time it is valuable as an anti-periodic. It is an active stimulant, and combined with some of the salts of the cinchona, one-half the ordinary dose of the latter will have a better effect than a full dose uncombined.

When taking polygonum, the patient feels it to be a stimulant, and will say, "that medicine warms me all over, I feel it to the tips of my fingers." In dysentery, when there is tormina and tenesmus, I give from m. xx to ʒi in mucilaginous drinks every hour or two, as occasion demands; and when the discharges are so frequent as to prostrate and cause loss of sleep, an enema of polygon., ʒi in an ounce of cold water, after each dejection, will calm and still the bowels in a short time. In hæmorrhage of the bowels, no drug within my knowledge given by the mouth and as enema will so soon check it. I think it has no, or but little, influence in reducing the rates of the pulse. I use it in all forms of bowel complaints, particularly in the diarrhœa of typhoid fevers. It acts as a diaphoretic and mild diuretic.

For the watery diarrhœa for children I use—

℞. Tinct. polygon., ʒi;

Tr. rhœi comp.;

Tinct. zinziber, aa ʒiv;

Tr. camphor, ʒij;

Sys. annise, ʒvi;

Sodæ bicarb., ʒi.

S. From half to a teaspoonful, according to good judgment. This formula having no opium in it is a safe domestic remedy.

Some of the fluid extracts are of little value. I think from want of care in gathering the true plant. There is a variety of the species known as heartsease; the leaf is larger than the true, marked with a dark blotch, which the true has not. The flower of the true is pink, that of the false white. The plant should be gathered when in bloom, and dried quickly in the house. Keep in tight paper bags, otherwise it soon looses its virtues.—*Med. Bi-Weekly.*

